# Retraction Note to: Circ_0008035 contributes to cell proliferation and inhibits apoptosis and ferroptosis in gastric cancer via miR-599/EIF4A1 axis

**DOI:** 10.1186/s12935-021-02122-4

**Published:** 2021-08-09

**Authors:** Chang Li, Yuan Tian, Yun Liang, Qingchun Li

**Affiliations:** 1 Department of Gastrointestinal Colorectal and Anal Surgery, China-Japan Union Hospital of Jilin University, No.126, Xiantai Street, Changchun, 130031 Jilin China; 2 Center of Physical Examination, China-Japan Union Hospital of Jilin University, Changchun, 130031 Jilin China

## Retraction to: Cancer Cell Int (2020) 20:84 https://doi.org/10.1186/s12935-020-01168-0

The Editors-in-Chief have retracted this article. Concerns have been raised regarding a number of figures, specifically:

- Figure 2e: the HGC-27 cyclin D1 panel appears to be identical to the AGS PCNA panel of Fig. 2f.
- Figure 2e: the HGC-27 PCNA panel appears to be identical to the AGS cyclin D1 panel of Fig. 2f.
- Figure 2e: the HGC-27 GAPDH panel appears to be identical to the AGS GAPDH panel of Fig. 2f.

In addition, the authors have not provided documents confirming that ethics approval was obtained for this study. The Editors-in-Chief therefore no longer have confidence in the reliability of the data reported in the article.

None of the authors have responded to any correspondence from the publisher about this retraction.

